# Dietary medium chain triglycerides for management of epilepsy: New data from human, dog, and rodent studies

**DOI:** 10.1111/epi.16972

**Published:** 2021-06-25

**Authors:** Felicity Y. Han, Lisa Conboy‐Schmidt, Galena Rybachuk, Holger A. Volk, Brian Zanghi, Yuanlong Pan, Karin Borges

**Affiliations:** ^1^ Faculty of Medicine School of Biomedical Sciences University of Queensland St. Lucia Queensland Australia; ^2^ Research and Development Nestlé Purina PetCare St. Louis Missouri USA; ^3^ Technical Communications Nestlé Purina PetCare EMENA Barcelona Spain; ^4^ Department of Small Animal Medicine and Surgery University of Veterinary Medicine Hanover Germany

**Keywords:** energy metabolism, energy storage, epilepsy, glucose, ketogenic diets, medium chain triglycerides, mitochondria, nonketogenic diets, seizure

## Abstract

Many studies show that glucose metabolism in epileptic brain areas can be impaired. Energy is crucial to maintain normal brain function, including ion and neurotransmitter balances. Energy deficits can lead to disruption of ion gradients, which can trigger neuronal depolarization and generation of seizures. Thus, perturbed metabolic processing of glucose in epileptogenic brain areas indicates a specific nutritional need for people and animals with epilepsy, as they are likely to benefit from auxiliary brain fuels other than glucose. Ketogenic diets provide the ketone bodies acetoacetate and β‐hydroxybutyrate, which can be used as auxiliary fuel by the brain. In approximately 50% children and adults with certain types of epilepsy, who can tolerate and maintain these dietary regimens, seizure frequency can be effectively reduced. More recent data demonstrate that addition of medium chain triglycerides (MCTs), which provide the medium chain fatty acids octanoic and decanoic acid, as well as ketone bodies as auxiliary brain energy, can be beneficial in rodent seizure models, and dogs and humans with epilepsy. Here, this evidence is reviewed, including tolerance in 65% of humans, efficacy studies in dogs, possible anticonvulsant mechanisms of actions of MCTs, and specifically decanoic acid as well as metabolic and antioxidant mechanisms. In conclusion, MCTs are a promising adjunct to standard pharmacological treatment for both humans and dogs with epilepsy, as they lack central nervous system side effects found with current antiepileptic drugs. There is now a need for larger clinical trials in children, adults, and dogs to find the ideal composition and doses of MCTs and the types of epilepsy that respond best.

**Key Points**

- In epileptogenic brain areas, energy metabolism can be impaired, and fuels other than glucose and independent from PDH activity are indicated to provide energy
- MCTs provide medium chain fatty acids (MCFAs) and ketones as auxiliary brain energy and can reduce seizures in rodent seizure models, and dogs and humans with epilepsy
- Also, MCFAs and ketones improve brain mitochondrial functions and antioxidant defense
- With some promising results in multiple studies in canine epilepsy, larger clinical trials of MCTs are needed in people to determine the level of efficacy in epilepsy
- Further research will help determine the compositions and doses of MCTs, as well as the types of epilepsy in children, adults, and dogs that respond best

## 1 INTRODUCTION

Although many antiseizure drugs (ASDs) are available, approximately one third of epilepsies in people as well as dogs are classified as drug resistant. This means there is inadequate seizure control despite being treated with two or more appropriately chosen ASDs.^1^, ^2^ In addition, many ASDs have side effects. Thus, there is an urgent need for new treatments.

Here, we review the existing knowledge about impairments in energy metabolism in epileptogenic brain areas in animal models, and dogs and humans with epilepsy. We summarize the anticonvulsant effects of medium chain triglycerides (MCTs) and medium chain fatty acids (MCFAs) in rodents, dogs, and humans and then discuss proposed anticonvulsant mechanisms.

## 2 ENERGY METABOLISM IN EPILEPSY

### 2.1 Glucose transport and cytosolic metabolism

Normally, in a fed state, glucose is the primary energy source of the brain. Lacking storage capacity, the brain requires a continuous supply of glucose, consuming more than 20% of body glucose in humans.^3^, ^4^ In the cytosol, glucose is broken down by glycolysis to generate two pyruvate, two adenosine triphosphate (ATP), and two nicotinamide adenine dinucleotide (NADH) molecules. When pyruvate enters mitochondria and subsequently the tricarboxylic acid (TCA) cycle, it can produce a net total of one ATP, one FADH_2_, and four NADH molecules. A maximum of two ATP molecules can be produced from FADH_2_ and three ATP molecules from NADH via the electron transport chain. Thus, the TCA cycle generates the majority of ATP during oxidative metabolism in mitochondria, whereas little ATP is produced from glycolysis in the cytosol.

**FIGURE 1:**
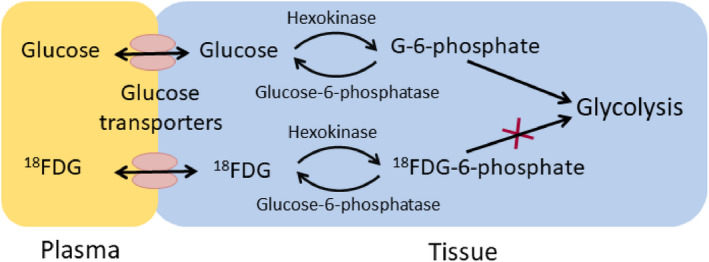
Simplified diagram of ^18^fluoro‐2‐deoxyglucose (FDG) metabolism. Similar to glucose, FDG is moved from plasma into tissue and cells by glucose transporters found on endothelial as well as neuronal and glial cells. Once inside the cells, again like glucose, FDG is phosphorylated by hexokinase to FDG‐6‐phosphate. However, subsequent processing of FDG‐6‐phosphate does not proceed along the lines of glucose‐6‐phosphate (G‐6‐phosphate) in the glycolytic pathway due to the lack of one hydroxyl moiety. Thus, FDG‐6‐phosphate remains mostly trapped inside the cells

The most common technique to quantify local cerebral glucose utilization (cerebral metabolic rate of glucose) is positron emission tomography with the radioactive glucose analogue ^18^fluoro‐2‐deoxyglucose (FDG).^5^, ^6^ Like glucose, FDG is taken up by the brain via facilitative glucose transporters and is phosphorylated by hexokinase. Thereafter, due to the lack of one hydroxyl moiety, FDG‐6‐phosphate gets largely trapped, as it is not further metabolized by glycolysis (Figure 1). The level of radiation emitted from FDG and its metabolites is thought to indicate the rate of glycolysis, because hexokinase is the rate‐limiting enzyme for glycolysis. Thus, low signals seen when using FDG positron emission tomography (PET) are interpreted as low glucose utilization and/or “hypometabolism,” which typically correlate with morphological abnormalities in epileptogenic brain areas.

Similar results with FDG‐PET and related methods have been found in the brains of people with epilepsy,^7^, ^8^, ^9^, ^10^, ^11^, ^12^ rodent epilepsy models,^13^, ^14^, ^15^ and the canine epileptic brain.^16^ Typically, in the interictal period, FDG‐PET signals from epileptogenic brain areas are lower than from healthy counterparts even if there is no damage in these areas,^17^ which suggests that impairments in brain glucose metabolism are possible. In human medicine, FDG‐PET has been used to localize epileptogenic foci to evaluate whether surgical removal of these areas is feasible. In the lithium pilocarpine rat model of temporal lobe epilepsy, FDG‐PET showed normal metabolism in the latent period of the model before spontaneous seizures occur, but limbic hypometabolic areas developed in the chronic “epileptic” period.^13^ Other rodent epilepsy models also showed low signals derived from FDG or other traceable glucose analogues, including ^14^C‐deoxyglucose and ^13^C‐glucose‐6‐phosphate in areas associated with seizure behavior.^14^, ^15^, ^18^ In dogs, FDG‐PET is emerging to become a more commonly used tool to diagnose epilepsy.^19^ As in people, in Finnish Spitz dogs FDG‐PET was superior to surface electroencephalography to localize epileptic foci.^16^

### 2.2 Mitochondrial metabolism

Once pyruvate enters the mitochondria, it is typically converted to acetyl‐CoA by the mitochondrial enzyme pyruvate dehydrogenase (PDH) and enters the TCA cycle. The glucose‐derived carbons provide the intermediates of the TCA cycle, which are also precursors for the biosynthesis of lipids, amino acids, and neurotransmitters. These precursors are important for healthy turnover of proteins and lipids contributing to structural components of the brain and repair of damage after seizures or other detrimental impacts. In addition, most of the ATP produced during aerobic metabolism is produced via oxidative phosphorylation of glucose‐derived carbons involving the TCA cycle, the electron transport chain, and ATP synthetase, also called Complex IV.

**FIGURE 2:**
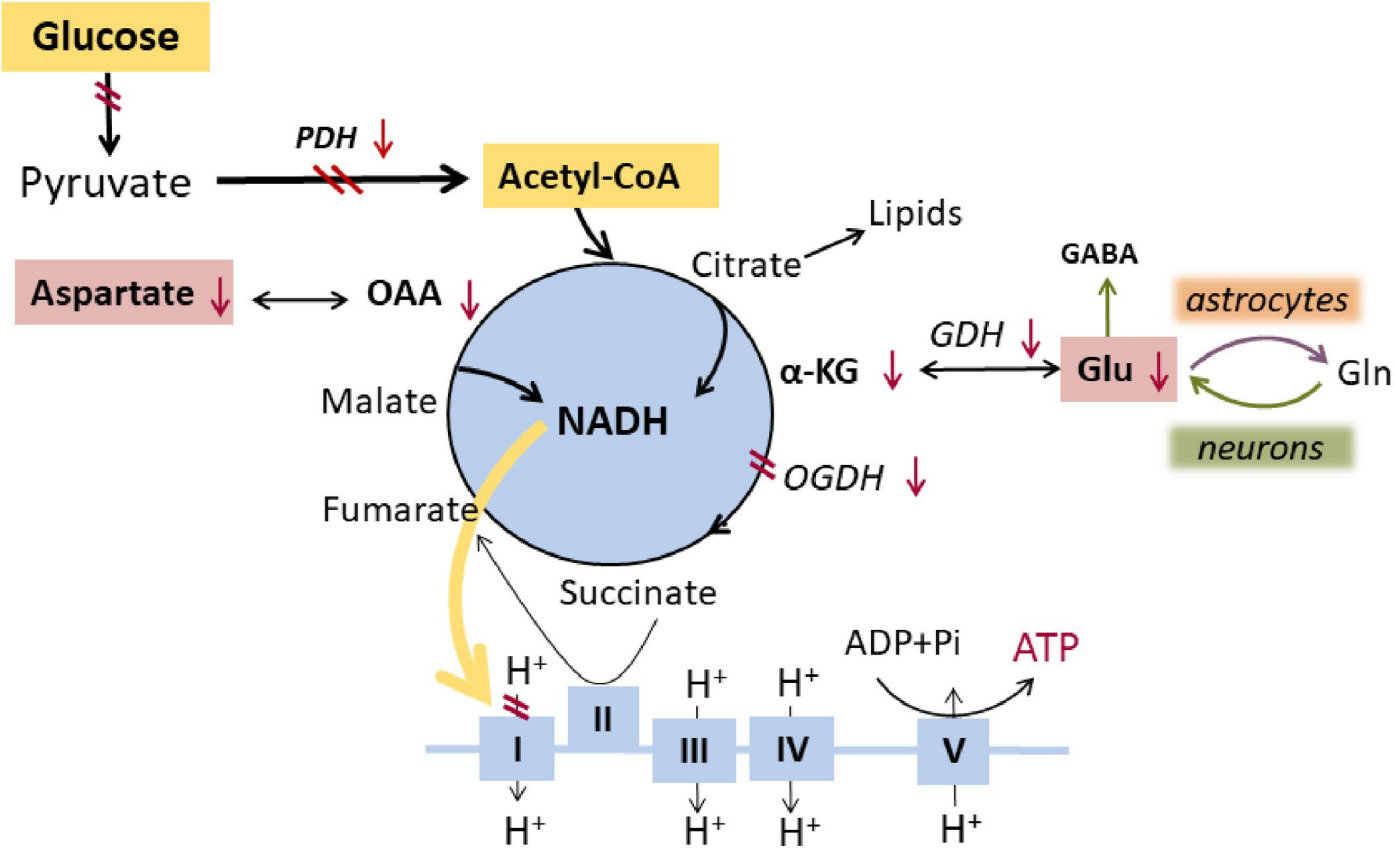
Changes in brain cell energy metabolism in epilepsy. Normally, oxidative glucose metabolism in mitochondria generates the most adenosine triphosphate (ATP) in brain cells, namely via the tricarboxylic acid cycle and the electron transport chain. Here, we show perturbations found in epileptogenic areas in rodent models, and dogs and people with epilepsy. Red arrows and double lines indicate reductions in metabolite levels, activities of pathways, enzymes or enzyme complexes, or glucose utilization. All these impairments can lead to reduced ATP production. ADP, adenosine diphosphate; α‐KG, α‐ketoglutarate also called 2‐oxoglutarate; GABA, γ‐aminobutyric acid; GDH, glutamate dehydrogenase; Gln, glutamine; Glu, glutamate; NADH, nicotinamide adenine dinucleotide; OAA, oxaloacetate; OGDH, oxoglutarate dehydrogenase; PDH, pyruvate dehydrogenase, Pi‐inorganic phosphate

We recently summarized findings regarding oxidative energy metabolism in epilepsy (Figure 2).^20^ In several rodent chronic epilepsy models, there were shortages of glucose‐derived intermediates and metabolites of the TCA cycle,^21^, ^22^, ^23^, ^24^, ^25^, ^26^ which correlated with reduced PDH activity in extracts from hippocampal tissue from mice with spontaneous seizures.^26^, ^27^ This is likely to result in deficits in the production of ATP via oxidative phosphorylation and a decreased ability for amino acid and lipid turnover, including glutamate–glutamine shuttling.^20^, ^28^ In addition, in people with epilepsy, impaired oxidative glucose metabolism, mitochondrial dysfunction, and mutations within mitochondrial constituents have been described,^29^, ^30^, ^31^, ^32^, ^33^ which can also add to energetic imbalances contributing to seizures.

Functional and structural changes in the electron transport chain described in humans and rodent models of epilepsy are typically accompanied by oxidative stress (for review, please see Pearson‐Smith and Patel,^34^ Rowley and Patel,^35^ Kovac et al.,^36^ and Rowley et al.^34^, ^35^, ^36^, ^37^). Deficiencies of Complex I activity were found in people with temporal lobe epilepsy, which is usually considered a consequence of oxidative stress.^38^ This finding coincided with an increased activity of succinate dehydrogenase staining in CA3 neurons, which may indicate that cells are trying to compensate for Complex I deficiency and produce more energy by adding more substrate into Complex II. These studies also support the theory that oxidative stress may cause reduced TCA cycle enzyme activity, because many of these enzymes are particularly sensitive to oxidation due to their structure.

Overall, the available data suggest that brain energy metabolism is impaired in epileptogenic foci. This includes (1) impairments in glycolysis from plasma‐derived glucose; (2) decreased activity of the TCA cycle with reduced entry of glucose‐derived carbons, which at least in part appears to be due to decreased activity of PDH; and (3) decreased production of biosynthetic precursors of amino acids and lipids, which may affect structural integrity and repair in brain tissue. More details of additional studies have been summarized by McDonald et al.^20^

### 2.3 Proposed mechanism of seizure generation via energy shortage

The lower signals seen with the glucose analogues, FDG, and related molecules in epileptogenic brain areas indicate that there is lower glucose transport and phosphorylation of plasma‐derived glucose by hexokinase . However, areas with epileptic activity require a large amount of energy, as there is high electrical activity, which can be recorded as “interictal spikes”—extra high neuronal activity between seizures. Thus, based on reduced use of plasma glucose, it is likely that there will be an energy deficit. Most of the energy in the brain is required to maintain ion and neurotransmitter balance during synaptic signaling, and also nonsignaling states of the brain, largely involving the energy‐dependent sodium potassium pump (also called sodium potassium ATPase) and glutamate transporters.^3^, ^39^, ^40^, ^41^ This is crucial to keep neuronal membrane potentials stable and synaptic activity under control. Thus, it is likely that energy deficits will reduce the activity of the sodium potassium pump and/or other important homeostatic transporters, such as glutamate transporters, which can contribute to neuronal hyperexcitability and subsequent seizure generation. This is corroborated by the finding that chronic inhibition of brain glycolysis (“chronic hypometabolism”) using 2‐desoxyglucose induced epileptiform activity in rats.^42^ Also, many genetic metabolic disorders are associated with epileptic seizures,^43^ including in people with deficiency of the main glucose transporter 1 (GLUT1), the transporter responsible for glucose uptake into the brain.^44^ Finally, data demonstrating the successful management of epilepsy in people, dogs, and rodents by using nutritional approaches, such as diets resulting in ketone body production, such as ketogenic and MCT diets, indicate that certain types of epilepsy may be caused by dysfunctions in energy metabolism.

**FIGURE 3:**
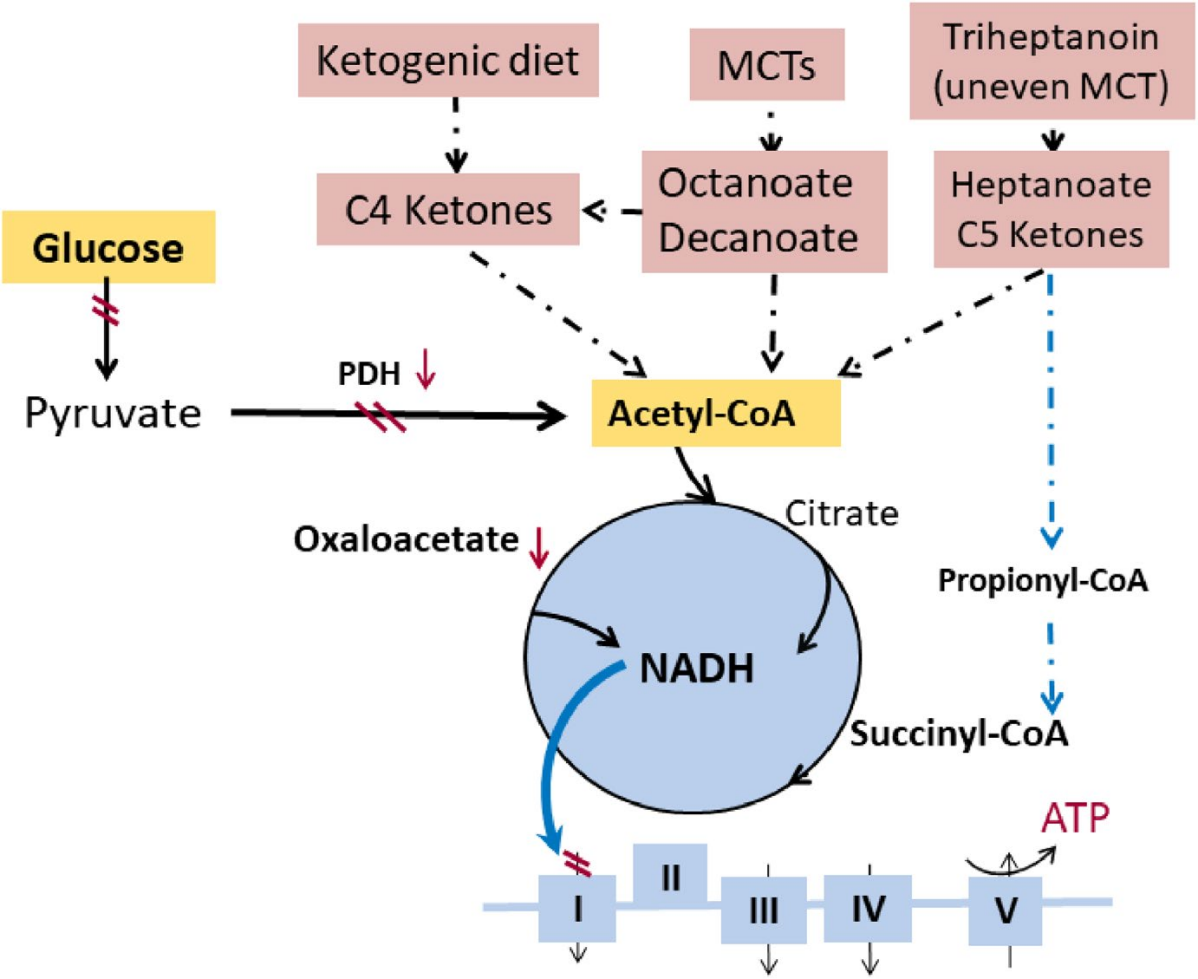
Auxiliary and alternative fuels and carbon sources for the brain. Several metabolic management approaches for epilepsy are highlighted with pink background, and their metabolic effects are indicated. This includes C4 ketone bodies, medium chain fatty acids (e.g., decanoate and octanoate), heptanoate, and C5 ketone bodies, which all directly produce acetyl‐CoA and do not require pyruvate dehydrogenase (PDH) for entry into the tricarboxylic acid (TCA) cycle. In addition, heptanoate and C5 ketone bodies also provide C4 TCA cycle intermediates by succinyl‐CoA. This anaplerosis may contribute to TCA cycling as well as biosynthesis of lipids and amino acids from TCA cycle intermediates. ATP, adenosine triphosphate; MCT, medium chain triglyceride; NADH, nicotinamide adenine dinucleotide

Taken together and as pointed out by a recent review with our collaborators, glucose hypometabolism found in epileptogenic brain areas provides the rationale for use of auxiliary fuels to provide energy for the brain to maintain normal neuronal signaling and to prevent the generation of seizures.^20^ Ideally, these fuels would enter the TCA cycle directly and bypass the enzymatic reactions of PDH that seem to be impaired. Thus, ketone bodies or MCFAs are good options (Figure 3), as their metabolism is independent of PDH activity and they can be supplied via the diet (see Sections 4 and 5).^20^ Consistent with this notion, the current standard treatment for GLUT1 deficiency in people is ketogenic diet,^44^ which provides ketone bodies that are used as fuel by the brain (e.g., Courchesne‐Loyer et al.^45^). Please note that lactate can also be used as an auxiliary fuel by the brain,^46^ but the generation of energy from lactate requires PDH activity. Also, to our knowledge, there is no suitable dietary way to provide lactate in high amounts, as the body cannot be overloaded with sodium or acid.

Overall, the available data suggest that brain energy metabolism is impaired in the epileptic brain and auxiliary fuels, other than glucose and independent from PDH activity, are needed to provide energy to epileptogenic brain areas. This includes ketone bodies, but also octanoic and decanoic acids, which are MCFAs that can be derived from MCTs—glycerol esters typically containing MCFAs with a chain length of between seven and 12 carbons.^47^ Triheptanoin, the triglyceride of heptanoic acid (C7), is an unusual specific MCT, as in nature it only occurs in very small amounts in certain seeds. In addition to providing fuel for the TCA cycle, triheptanoin can also provide succinyl‐CoA and refill the intermediates of the TCA cycle (anaplerosis).^48^ Data about triheptanoin treatment for epilepsy in rodent models and clinical trials can be found elsewhere.^20^, ^49^, ^50^, ^51^, ^52^, ^53^, ^54^

## 3 KETOGENIC DIETS AND MCTs: FORMULATIONS AND BIOCHEMISTRY

In the 1920s, medical ketogenic diets that increase the plasma levels of C4 ketone bodies were invented to mimic the beneficial effects of fasting for the treatment of epilepsy in children (reviewed in Huttenlocher et al.^55^, ^56^). Ketogenic diets used for the management of epilepsy are typically very high in fat (originally four parts of weight), but very low in protein and carbohydrates (one part in weight together) and are often calorically restricted. In response, blood glucose levels remain in the lower normal range and the liver uses fatty and amino acids to produce the C4 ketone acetoacetic acid and β‐hydroxybutyric acid. Under these conditions, rodent and human brains use these ketone bodies as fuel and less glucose.^45^, ^57^, ^58^ The canine brain can also use ketone bodies as fuel, as shown during hypoxia and infusion of β‐hydroxybutyrate.^59^ In the 1970s, to allow patients to consume higher amounts of carbohydrates and protein, the MCT ketogenic diet was developed.^47^, ^55^ In the original version, 50%–65% of energy intake is from MCTs, but another version only uses 30% MCTs.^60^ The added MCTs release MCFAs, mostly octanoic and decanoic acids, which in the liver can quickly be turned into ketone bodies. Especially MCTs enriched in octanoate are well known to increase ketone body production (e.g., see Pierre et al.^61^). Another version of the original ketogenic diet is the modified Atkins diet, which allows reducing the large amounts of fats. Currently, versions of ketogenic‐derived diets use different fat versus carbohydrate/protein ratios, such as 3:1 and 2:1. Two recent meta‐analyses of many clinical prospective studies reported approximately 50%–65% efficacy of these versions of original and modified ketogenic diets in the treatment of various adult and childhood epilepsies.^56^, ^62^, ^63^ Please note that in children with certain types of epilepsy, the MCT‐ketogenic diet seems to be similarly effective as the classical ketogenic diet in regard to seizure control.^64^, ^65^, ^66^ Moreover, the use of ketogenic diets and MCTs is expanding to other neurological disorders, including Alzheimer disease, Parkinson disease, and mild cognitive impairments.^67^, ^68^, ^69^, ^70^ In addition to the provision of C4 ketone bodies as fuel, ketogenic diets are also thought to promote mitochondrial biogenesis, activate protective transcription factors such as peroxisome proliferator‐activated receptor‐γ2 (PPARγ2), and have antioxidant and anti‐inflammatory effects, which together appear to limit seizure generation.^71^, ^72^, ^73^ Please note that high fat ketogenic diets are difficult to follow for families and especially adults with epilepsy, as many everyday snacks and treats are not allowed.

MCTs added to a regular diet containing carbohydrates appear to be a new advantageous option to provide auxiliary brain fuel. Commercially available MCTs, which are used by people generally interested in well‐being, typically contain 30%–70% octanoic and 30%–70% decanoic acids (C8 and C10 fatty acids, also called caprylic and capric acid, respectively). Being tasteless for people, MCTs can easily be added to meals or intragastric/duodenal nutrition and provide energy quickly. To increase tolerability, they can be emulsified with milk^68^ or powdered. MCTs in the form of oils, powders, drinks, and nutrition bars are also used as dietary supplements by athletes and people with metabolic or digestive problems or while on ketogenic therapy. In addition, some people use MCTs to increase satiety and curb appetite to lose weight or avoid gain of body weight.^47^, ^74^ After hydrolysis of MCTs in the gastrointestinal tract by lipase, the free MCFAs can diffuse directly into portal vein and then mostly to mitochondria of liver, but also extrahepatic tissues.^47^ In the brain, MCFAs are mostly metabolized by astrocytes (see below). In contrast, long chain fatty acids are more slowly metabolized, because they are first transported by the lymph and require transport proteins in the blood and for final transport into mitochondria for β‐oxidation. Also, in the brain, there is limited metabolism of long chain fatty acids for the derivation of energy.^75^ As mentioned above, octanoate and to a lesser extent decanoate are converted by the liver to C4 ketone bodies.

Data from rodents indicate that MCFAs diffuse into the brain.^76^ Labeling of octanoic and heptanoic acids with the carbon isotopes ^11^C, ^14^C (both radioactive), or ^13^C showed that after injection, these MCFAs instantaneously appeared in the brain and were immediately metabolized.^53^, ^77^ Because the labeled carbons were mostly found in brain glutamine, and astrocytes specifically contain glutamine synthetase and produce glutamine in the brain, it is likely that in vivo mostly astrocytes metabolize MCFAs.^53^, ^77^, ^78^ In rats, the infusion of labeled octanoic acid for 105 min resulted in plasma concentrations of 250 µmol·L^–1^ ^13^C‐octanoate,^77^ levels that are also achieved by oral ingestion of medium doses of MCTs. Modeling showed that octanoate provided 20% of brain energy.^77^ To our knowledge, there are no in vivo studies regarding the metabolism of decanoic acid, and solubility of decanoic acid is limited. Thus, injection of sufficiently high amounts to allow detection of brain decanoate metabolites is currently not possible. Similar to ketone bodies, MCFAs can enter the TCA cycle without requiring PDH activity. This indicates that MCFAs could directly supplement the energy needs in epileptogenic brain areas, where two studies have found reduced PDH activity (see above and Figure 3).^26^, ^27^ Taken together, several studies indicate that MCTs and MCFAs provide auxiliary fuel and can alter brain energy metabolism. Several recent studies have shown anticonvulsant effects after adding MCTs to regular diets in rodents, dogs, adults, and children, as summarized in the next paragraphs.

## 4 ANTICONVULSANT EFFECTS OF MCTs

### 4.1 Rodents

Anticonvulsant effects of MCFAs and MCTs of various compositions have been observed in several acute rodent models (Table 1), which can predict activity for different types of human seizures.^79^ In the 6‐Hz mouse model, a model thought to reveal efficacy of drugs against focal unaware seizures, anticonvulsant effects were seen 30 min after acute gavage with octanoic and decanoic acid (10–30 mmol·kg^–1^, approximately 1.4–5.2 g/kg)^80^, ^81^ and after 10 days of feeding 35E% (% of energy) MCTs containing 95% decanoic acid (tridecanoin or tricaprin).^82^ In the first experiment, we also found an anticonvulsant effect in the 6‐Hz model with feeding 35E% trioctanoin (tricaprylin), the triglyceride of octanoic acid.^83^ This effect could not be reproduced later, which remains unexplained, but has happened before with metabolic treatments.^84^ In the intravenous pentylenetetrazol model, a model likely to show efficacy of treatments against generalized unaware seizures in people, only octanoic acid increased the seizure threshold. In contrast, using flurothyl, another γ‐aminobutyric acid type A (GABA_A_) receptor channel blocker, only tridecanoin but not trioctanoin was effective.^82^ In the maximal electroshock model, a model typically indicating efficacy of treatments against acute tonic–clonic seizures, only high amounts of decanoic acid (50 mmol·kg^–1^ or 8.5 g/kg) were effective, but not octanoic acid.^80^, ^81^ The feeding studies with MCTs were done in the last author's laboratory and used 35E% of MCTs, which corresponds to approximately 180 g of MCTs per kilogram of mouse chow. Adult CD1 mice have been estimated to eat 3 g of chow per day and thus would have consumed approximately 18 g of MCTs per kilogram of body weight per day. Thus, the anticonvulsant doses of MCFAs (10–30 mmol·kg^–1^, approximately 1.4–5.2 g/kg) given in the acute oral gavage studies are in a similar range to the 35E% MCT feeding studies.

**TABLE 1:** Single medium chain fatty acid MCTs, triheptanoin, octanoic, and decanoic acid in acute mouse seizure models

Models	Oral gavage		Triglycerides (35E%) mixed in mouse chow		
	CA8^80^	CA10^147^	Trioctanoin (C8)^82^, ^83^	Tridecanoin (C10)^82^	Triheptanoin (C7)^24^, ^148^
6 Hz	Yes at 10–30 mmol·kg^–1^	Yes at 10 and 30 mmol·kg^–1a^	Inconsistent	Yes	Inconsistent
Maximal electroshock	No effect at 5–30 mmol·kg^–1^	Yes at 50 mmol·kg^–1^	N.D.	N.D.	Yes
Pentylenetetrazole (i.v.)	Yes at 10–30 mmol·kg^–1^ for myoclonic and clonic convulsions No for tonic convulsions	No at 10–50 mmol·kg^–1^	N.D.	N.D.	Yes for tonic seizures
Flurothyl	N.D.	N.D.	No	Yes for first generalized seizure and tonic extension	Inconsistent

Abbreviations: CA8, caprylic acid (MW = 144); CA10, capric acid (MW = 172; also called decanoic acid); E%, % of energy; i.v., intravenous; MW, molecular weight; N.D., not determined.

^a^ Similar effect when 20 mmol·kg^–1^ CA8 was added to 30 mmol·kg^–1^ CA10.

In two ex vivo rat hippocampal slice models, 1 mmol·L^–1^ decanoic acid, but not octanoic or nonanoic (C9) acids, completely prevented epileptiform discharges induced by pentylenetetrazol or low magnesium as measured with field electrodes.^85^, ^86^ The effects of this high decanoic acid concentration were attributed to α‐amino‐3‐hydroxy‐5‐methyl‐4‐isoxazolepropionic acid (AMPA) type glutamate receptor inhibition^86^ and may also explain why with gavage of approximately 100 mmol (17 g) decanoic acid/kg, 50% of mice show motor impairments in the chimney test.^81^ On the other hand, no motor impairments have been seen in humans or dogs using MCTs (see Sections 5.2 and 5.3). Thus, the contribution of AMPA receptor inhibition when using MCTs to manage epilepsy requires more research (see further discussion in 5).

In summary, octanoic and decanoic acids and their MCTs showed anticonvulsant activities in rodent models of acute seizures. The effects of octanoic versus decanoic acids and their MCTs were mostly similar in the 6‐Hz model and comparable to the magnitude of effects of ketogenic diets.^84^, ^87^ This is important because new ways to manage focal unaware seizures in people are most urgently needed, as these types of seizures are often treatment resistant. Small or no effects of these MCFAs and MCTs were seen in the maximal electroshock test and the models using the GABA_A_ receptor channel blockers pentylenetetrazol and flurothyl. This indicates that the anticonvulsant effects and mechanisms of MCFAs are at least partially distinct, with different carbon chain lengths and MCT versus MCFA administration. Potential anticonvulsant mechanisms are discussed in Section 5. It is also important to bear in mind that all these rodent studies used healthy “normal” animals without any known brain energy deficits and the magnitude of the effects were small when compared to ASDs (e.g., see the PANAChE database, https://panache.ninds.nih.gov/). To our knowledge, there are no studies in chronic epilepsy models in rodents, which are now required before new pharmaceutical treatments enter clinical trials. Unlike the mostly pure octanoic or decanoic acid formulations employed in these published rodent studies, for people and dogs, MCTs that contain mixtures of octanoate and decanoate are used. Mixed MCTs are better tolerated than pure trioctanoin (Stephen Cunnane, personal communication), and unlike tridecanoin, they remain in oil form at room temperature. Oils are more easily measured and mixed into food than solids, and production of powders and shakes is more straightforward.

### 4.2 Dogs

In dogs, specific breeds in certain countries have shown very high prevalence of epilepsy. This includes, but is not limited to, certain Belgian shepherd populations in Denmark and US Irish wolfhounds, with 33% and 18% of dogs suffering from epilepsy, respectively (reviewed in Hülsmeyer et al.^88^). In 2018, data from veterinary primary care practices in the UK showed that pugs, boxers, basset hounds, border terriers, and border collies had a higher prevalence of epilepsy (1.5%–1.9%) compared to other breeds.^89^ The onset of epilepsy typically occurs in the first few years of life.^89^ In addition to epileptic seizures, dogs with epilepsy show behaviors similar to anxiety and attention‐deficit/hyperactivity disorder in humans, as well as cognitive impairments.^90^, ^91^, ^92^ Typically, older ASDs are used to treat epilepsy in dogs.^88^, ^93^ This includes phenobarbital (an allosteric modulator of the GABA_A_ receptor), imepitoin (a low affinity partial agonist for the benzodiazepine binding site of the GABA_A_ receptor), and potassium bromide, where bromide is thought to hyperpolarize neurons by replacing chloride. These older treatments commonly have side effects, such as sedation, ataxia, and polyphagia.^94^ Levetiracetam, zonisamide, and other newer antiepileptic drugs used in people are also used in dogs with variable success and are expensive.^2^ Similar to humans, the side effects from ASDs in dogs often reduce quality of life and can be life‐limiting, due to blood dyscrasias and liver toxicity.^94^, ^95^, ^96^

Safety regarding the feeding of MCTs was studied in dogs for 90 days, and 5%, 10%, and 15% MCTs (wt/wt) were found to be safe.^97^ A recent study using metabolomics of serum^98^ also showed that the feeding of a commercial diet enriched with 5.5% (wt/wt) MCTs to dogs with epilepsy did not adversely alter blood metabolic parameters compared to those dogs given a standard commercial diet. There were no issues regarding tolerability and palatability of MCTs at the tested inclusion rates in dogs.

To date, several efficacy studies in dogs with idiopathic epilepsy fed with 5.5%–6.5% added MCT oil (wt/wt) or MCT oil providing 9% of daily metabolic energy requirement have been published (Table 2). The ASD regimens of the dogs remained the same between the placebo and MCT diet phases. All dogs in these trials were considered drug‐nonresponders to standard ASDs, such as phenobarbital, potassium bromide, and levetiracetam. Two publications document the effects of feeding of commercial diets containing 5.5% MCT oil versus 5.5% lard in one cohort of dogs in a 3‐month randomized double‐blinded crossover study.^99^, ^100^ There were no severe side effects and no significant changes in body weights as well as serum concentrations of phenobarbital or potassium bromide between the placebo and MCT diet, indicating that the addition of MCTs to dog food did not affect the metabolism of these ASDs.^99^ Ten of 21 dogs (47%) had more than 50% reduction in seizure frequency, with three dogs becoming seizure‐free.^99^ Regarding changes in behavior, less stranger‐directed fear was observed among different behaviors noted, indicating potential anxiolytic effects of add‐on MCTs.^100^ A nonblinded study investigated the effects of a 6.5% MCT commercial diet in dogs with epilepsy and found a reduction in seizure frequency by 33% over a 3‐month period without changes in ASD blood levels.^101^ Nine of 21 dogs (43%) had more than 50% reduction in seizure frequency, including two dogs becoming seizure‐free.^101^ In a recently published multicenter randomized double‐blinded crossover study, dog owners were taught to add MCT oil (contributing 9% daily energy) containing 50%–65% octanoate and 30%–50% decanoate versus colorless, extra virgin olive oil to their dogs' regular food for a total of 28 dogs.^102^, ^103^ The case number chosen for this study was based on the results of Law et al.,^99^ who used a similar study design.^102^, ^103^ A power analysis was performed and indicated that 22 dogs would be sufficient using Wilcoxon signed ranked test to reach a power of .95 (90%) at Type I error rate of .05 (PASS V19.02, NCSS, Statistical Software). Overall, there was a significant 22% reduction in the median number of seizures with add‐on MCT feeding. Two dogs became seizure‐free, and three dogs had more than 50% reduction in number of seizures, corresponding to efficacy in 18% of dogs. In this study, cognition was also evaluated, showing that MCT supplementation improved spatial–working memory, problem‐solving ability, and owner‐reported trainability in dogs with difficult to treat epilepsy.^104^

**TABLE 2:** Anticonvulsant effects in dogs during three studies of MCTs included in a regular diet

Study type, amount of MCTs vs. placebo, wt/wt % or E%	Total number of dogs	Mean seizure frequency reduction, %	Dogs with >50% reduction in seizure frequency, *n* (%)	
			MCTs	Placebo
Blinded crossover study, 5.5E%–10E% MCTs vs. lard in a commercially produced diet^98^, ^99^	21	13.5%	10 (47%)	1 (5%)
Nonblinded 6.5% MCTs in a commercially produced diet^101^	21	33%	9 (43%)	
Blinded 9E% add‐on MCTs vs. colorless extra virgin olive oil added to “regular” dog food^103^, ^149^	28	22%	(18%)	2 (7%)

Abbreviations: E%, % of energy; MCT, medium chain triglyceride.

These studies are also complimented by a recent case report. One dog, which did not show seizure reduction with standard phenobarbital and bromide therapy and had weekly seizures, switched from a white fish/white potato diet to a modified Atkins type ketogenic diet containing 15% MCTs. The dog was reported to be seizure‐free for 33 weeks, except for two breakthrough seizures when not eating for 2 days in the absence of his owner.^105^

The magnitudes of the anticonvulsant effects in dogs associated with dietary MCT supplementation published^99^, ^101^, ^103^ are very similar to successful new ASDs in large human Phase III efficacy trials, where new drugs are tested in 200–600 patients with drug‐resistant epilepsy as add‐on treatment. In these trials, approximately 30%–50% of people experienced greater than 50% reduction in seizures when treatments were deemed efficacious, and the placebo showed efficacy in 10%–26% of people.^106^, ^107^, ^108^, ^109^, ^110^ On average, seizure frequencies were reduced by 26% with efficacious drugs and 13% with placebo.^106^, ^107^, ^108^, ^109^ Taken together, these studies provide accumulating evidence of the efficacy of MCTs as an adjunct for the management of epilepsy in dogs.

### 4.3 Humans

In 2013, a case report reported marked seizure reduction in a man who added four tablespoons of MCT oil twice per day to this regular diet.^111^ In a first small controlled randomized double‐blinded clinical trial to test the tolerability of add‐on MCTs versus triheptanoin in Melbourne with 34 adults with treatment‐resistant epilepsy, participants were asked to mix oils into three daily meals and titrate oil doses up slowly over several weeks to their maximal tolerated amount (maximal 35E% or 100 ml/day) while eating to satiety.^49^ The oils were tolerated in approximately two thirds of participants. Discontinuation was due mostly gastrointestinal problems, such as diarrhea, and in a few people increases in seizure activity. Side effects were mostly minor and mostly restricted to gastrointestinal upset, including diarrhea, stomach cramps, or constipation. This could be managed by slow titration, mixing with food, reducing doses, and dietitian counseling. In contrast to ASDs, no cognitive or other severe side effects were reported. A median body weight gain of 2 kg was found, although participants in the study were encouraged to eat to satiety and reduce normal food portions. Thus, it appears that, different from other populations, MCTs in this group of people with epilepsy did not reduce food intake. As people with epilepsy tend to be overweight,^112^ this is a potential concern for future studies. In the MCT arm, five of 11 (45%) adults finishing the study, all with focal unaware seizures, showed greater than 50% reduction in seizure frequency. These adults ingested 40–70 ml MCTs per day containing 55% octanoic and 45% decanoic acids (mean = .73 ml/kg body weight).^49^ After cessation of MCTs, seizures returned in all but one adult.

Anticonvulsant effects of add‐on MCTs in a normal diet were also seen with comparable amounts of a decanoic acid‐enriched MCT emulsion drink developed by Vitaflo in adults and children with treatment‐resistant epilepsy in a tolerability study (see Poster 3.367 at American Epilepsy Society Annual Meeting 2019).^113^ The outcomes of this randomized double‐blinded study in 61 people using a placebo oil emulsion drink were very similar to the Australian study in regard to tolerability, side effects, and efficacy measured as the reduction of the number of seizures in children and adults. This larger study in the UK did not report weight gain. Participants were told to reduce foods enriched with simple sugars. Thus, potential weight gain can be managed with dietetic counseling. Another option may be to combine MCTs with a low glycemic index diet, which aims to maintain consistent low to normal blood glucose levels. A low glycemic index diet also encourages food with complex carbohydrates and avoids high amounts of simple sugars and refined carbohydrates.

Taken together, these human clinical trials testing tolerability of MCTs as primary endpoints were small and not designed to testing efficacy. Moreover, tolerability was an issue for one third of patients. However, there is the possibility that when MCTs are tolerated, they may be a potential adjunct for the management of epilepsy in people. The lack of toxic and neurocognitive side effects encourages more research into MCTs. Based on the available studies, it is still unclear which MCT formulation is ideal to prevent seizure generation, although MCTs containing decanoic acid seem to be more efficacious in in vitro and in vivo rodent models. In the future, it will be necessary to find the most effective dose, the ideal ratio for octanoic versus decanoic acids, and the types of epilepsy that respond best. It will also be important to find MCT formulations that are better tolerated by humans than oils and emulsions, potentially powders or MCTs in certain food matrices (bars, shakes, etc) may be an option. A larger clinical trial of MCTs in adults with focal epilepsy is currently being prepared by Professor Seungbong Hong and the last author. The study is planned to start at the Samsung Medical Center in Seoul, South Korea. If promising, it can be expanded to include other Korean hospitals.

## 5 PROPOSED ANTISEIZURE MECHANISMS OF MCTs

### 5.1 Levels of MCT metabolites

In people and rodents, MCTs in regular diets provide 50–400 µmol·L^–1^ MCFAs in blood, while reports about increased levels of C4 ketone bodies vary and also depend on the type of MCT used.^61^, ^68^, ^82^, ^114^ Please note that ketone bodies and MCFAs can be metabolized very rapidly. Therefore, it is difficult to assess the turnover of these molecules when only measuring blood and tissue levels. Also, the levels found in blood are higher than those found in brain, which is consistent with fast metabolism in the brain.^82^ For example, using microdialysis in mice, the β‐hydroxybutyrate concentration in hippocampal extracellular fluid was found to be around 50 µmol·L^–1^,^115^ whereas blood levels were around 1.5 mmol·L^–1^ in the same mouse strain fed a 4:1 ketogenic diet.^87^ Opinions regarding biologically relevant C4 ketone body levels in blood vary, although most people tend to agree that levels greater than 400 µmol·L^–1^ β‐hydroxybutyrate in people are physiologically significant (Mary Newport, personal communication).

In rats, feeding 5% MCT diet increased blood β‐hydroxybutyrate levels modestly by up to approximately 50% from the 150 µmol·L^–1^ baseline.^116^ In mice, neither 35E% tridecanoin nor trioctanoin consistently increased ketone body levels in blood and brain of mice.^82^, ^83^ However, 30 min after acute gavage of 30 mmol·kg^–1^ decanoic acid, an elevation of β‐hydroxybutyrate from .57 mmol·L^–1^ to 1.66 mmol·L^–1^ was found in trunk blood together with 300 µmol·L^–1^ decanoate.^81^ Gavage of 20 mmol·kg^–1^ octanoic acid produced around 400 µmol·L^–1^ octanoate and increased β‐hydroxybutyrate from .28 mmol·L^–1^ to .9 mmol·L^–1^ in blood.^80^ Thus, acute loading with MCFAs seems to increase C4 ketone body levels more than chronic feeding.

In dogs fed with MCTs, the blood levels of β‐hydroxybutyrate showed a significant increase up to 120 µmol·L^–1^,^99^, ^117^ whereas MCFA concentrations have not been measured so far. In humans, plasma levels of octanoic, decanoic, and dodecanoic (C12) acids remained increased between 100 and 300 µmol·L^–1^ for 6–8 h when volunteers ingested two doses of 20 ml MCTs 4 h apart.^61^ The MCTs tested contained either largely octanoic, decanoic, or alternatively dodecanoic acids or a mixture of 55% octanoic and 35% decanoic acids. The largest levels in plasma C4 ketone bodies (200–800 µmol·L^–1^) were found with trioctanoin, followed by the MCTs containing both octanoic and decanoic acids (200–600 µmol·L^–1^) and tridecanoin (200 µmol·L^–1^). In our human randomized controlled trial with MCTs containing both octanoate (55%) and decanoate (45%), we found no increase in serum ketone levels when employing commercially available ketone meters used by people with diabetes, which have low sensitivity (>400 µmol·L^–1^ β‐hydroxybutyrate).^49^

All studies could benefit from evaluation of C4 ketone body and MCFA concentrations over time and ideally brain levels of MCT metabolites; however, the latter is possible only postmortem. On the other hand, blood and also brain levels are not always useful to appreciate fast turnover of fuel molecules. Therefore, in any case it will remain difficult to distinguish effects of MCFAs and their ketone body metabolites, as both can serve as auxiliary fuels and also have other additional nonmetabolic effects (see below).

### 5.2 MCFA metabolic effects in vivo and in vitro

In mice fed trioctanoin, amounts of several metabolites were changed in the hippocampus,^83^ consistent with reduced activity of phosphofructokinase found in hippocampal extracts.^82^ The levels of fructose 1,6 bisphosphate, dihydroxyacetone, malate, and succinate were lowered, whereas the levels of glucose‐6‐phosphate and fructose‐6‐phosphate were higher than in control diet‐fed mice. Interestingly, the concentrations of metabolites of the pentose phosphate pathway, ribulose 5‐phosphate and xylulose 5‐phosphate, were also low, suggesting that overall cytosolic glucose metabolism was reduced, which could be an effect of increased metabolism of MCFAs to produce energy.^83^

The results from these in vivo studies are consistent with data showing that cultured astrocytes derived from embryonic mouse brain and also human stem cells can metabolize MCFAs in vitro.^82^, ^118^, ^119^ However, a cultured neuronal cell line also oxidized MCFAs.^120^ The study in the neuronal cell line SH‐SY5Y showed that oxidation of octanoic acid was much faster and competed with that of decanoic acid. Octanoate oxidation was also less dependent on the mitochondrial enzyme carnitine palmitoyltransferase I (CPT1), based on its inhibition by the CPT1 inhibitor etoxomir.^120^ This finding is of interest as it indicates that a mixture of octanoate and decanoate in MCTs may be ideal to achieve high decanoate levels in tissue.

In contrast to the studies mentioned above, feeding a diet containing 5% MCTs with 40:60 of octanoate versus decanoate for 2 weeks reduced mitochondrial respiration and protein amounts of enzymes related to glycolysis and oxidative phosphorylation in the medial prefrontal cortex of anxious rats.^116^ On the other hand, proteins controlling glucose (GLUT1) and glutamate transport (glutamate transporter 1) were increased in this brain area.^116^ Similarly, in human induced pluripotent stem cell‐derived astrocytes, 100 to 300 µmol·L^–1^ MCFAs inhibited mitochondrial metabolism and reduced NAD(P)H fluorescence.^121^ Please note that the results from these two studies from the same institute are very different from results found in “healthy” rodents, epilepsy models, and cultured brain cells and may be specific to anxiety or other experimental conditions.

### 5.3 Other proposed anticonvulsant effects of MCFAs

**FIGURE 4:**
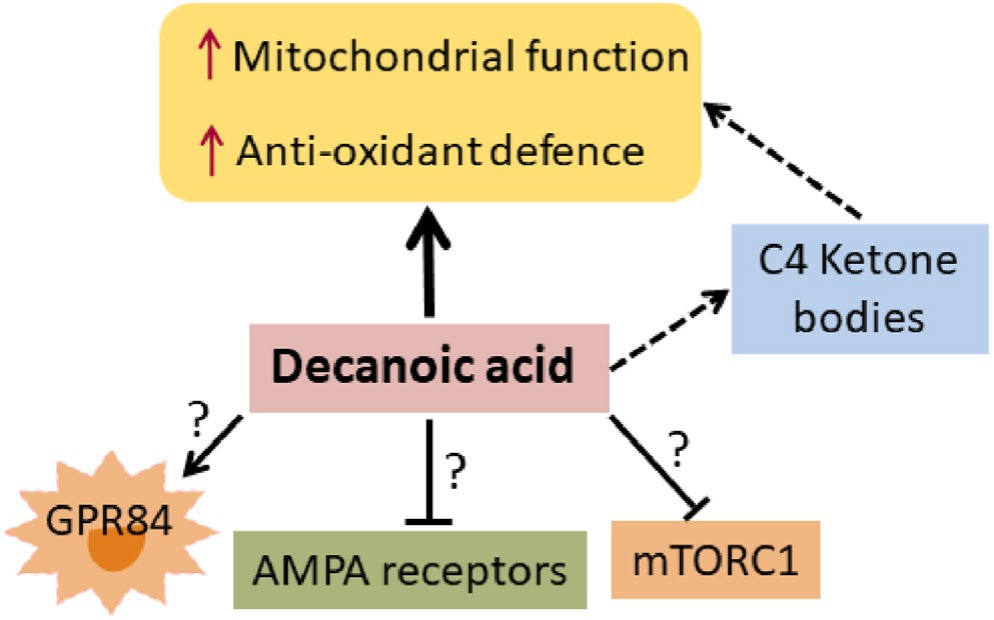
Simplified schematic diagram of nonmetabolic actions of decanoic acid that may contribute to its anticonvulsant effects. Several mechanisms of action have been suggested, including direct effects of decanoic acid on improvement of brain mitochondrial function and antioxidant defense found in vitro and in vivo. These effects were also found with ketogenic diets and could be at least in part mediated via the formation of C4 ketone bodies. Low amounts decanoic acid can activate GPR84, whereas high decanoic acid levels are needed for the inhibition of α‐amino‐3‐hydroxy‐5‐methyl‐4‐isoxazolepropionic acid (AMPA) type glutamate receptors in neurons and the mechanistic target of rapamycin complex 1 (mTORC1) signaling pathway. The contribution of these effects remains to be shown in vivo.

Apart from providing additional fuel, MCFAs and ketone bodies also have other nonmetabolic actions that may contribute to their anticonvulsant effects (Figure 4). Metabolism of ketone bodies in the brain may result in increased synthesis of the major inhibitory neurotransmitter, GABA, which can contribute to antiepileptic effects by reducing hyperexcitability of neurons.^122^ In addition, several groups reported improvement of brain mitochondrial function and antioxidant defense in response to ketogenic diets, MCTs, MCFAs, and ketone bodies in vivo and in vitro.^71^, ^72^, ^73^, ^82^, ^123^, ^124^, ^125^, ^126^ In mice fed 35E% tridecanoin, there was higher respiratory activity linked to ATP production in mitochondria isolated from hippocampal formations, which is expected to enhance brain energy reserves. In addition, a better ability to reduce ferric iron in blood coincided with increased expression of heme oxygenase 1 (Hmox1) and FoxO1 mRNA in brain.^82^ FoxO1 is a transcription factor that is known to upregulate Hmox1, an important Phase II antioxidant enzyme known to provide homeostatic redox defense in glial cells. In aged dogs, feeding 2 g MCT/kg body weight was also shown to increase mitochondrial function, namely it enhanced State III and Complex I‐driven respiration in the parietal cortex and reduced oxidative stress markers in mitochondria.^126^

Another potential antiseizure effect was shown in rats, which were given high oral amounts of MCFAs. The levels of tryptophan were found to be low in blood, but high in brain tissue.^127^ Also, low blood tryptophan levels have also been seen in children on ketogenic diet.^128^ In the brain, tryptophan may be metabolized to NAD^+^, which may affect energy metabolism. On the other hand, metabolism of tryptophan to NAD^+^ includes formation of the intermediate quinolinic acid, a neurotoxin.

In vitro, at very low concentrations (4.5 µmol·L^–1^), decanoic acid can activate the inflammatory receptor GPR84.^129^ This receptor is normally expressed at low levels, but is induced by the strong immune activator lipopolysaccharide. Expression is then mostly found in monocytes, neutrophils, and also in microglia in the brain.^130^, ^131^ However, the roles of this proinflammatory receptor regarding anticonvulsant or other effects are still unclear. No adverse effects regarding inflammation have been seen over the many years of using MCTs in children with epilepsy and people with metabolic disorders.

Higher amounts, namely 50 µmol·L^–1^ of decanoic acid and also other longer chain fatty acids, such as myristic and linoleic acid, can activate the transcription factor PPARγ2, which can promote mitochondrial biogenesis and has antioxidant effects.^82^, ^118^, ^132^, ^133^

When 250 µmol·L^–1^ decanoic acid was added to cultured cells, it increased mitochondrial proliferation via the PPARγ receptor and increased catalase activity in SH‐SY5Y cells.^123^ Neither increases in catalase activity^82^ nor higher mitochondrial DNA copy numbers were found in brains from tridecanoin‐fed mice in the last author's laboratory. However, increased brain catalase activity was also seen in mice fed ketogenic diet, which could be blocked with a PPARγ inhibitor.^73^, ^133^, ^134^ In human induced pluripotent stem cell‐derived astrocytes, 250 µmol·L^–1^ decanoic acid increased glutathione amounts,^119^ confirming antioxidant effects found in the other studies mentioned above.

In addition, at high concentrations (half‐maximal inhibitory concentration = 250 µmol·L^–1^), decanoic acid can act as a noncompetitive AMPA receptor antagonist, resulting in direct inhibition of excitatory neurotransmission, whereas octanoic acid was inactive.^86^ However, so far, no sedative or other central nervous system side effects have yet been observed in people and dogs (see Section 5.1). Also, a recent publication found that 300 µmol·L^–1^ decanoic acid can block the activity of mechanistic target of rapamycin complex 1 in ex vivo rat hippocampus and in tuberous sclerosis complex patient‐derived astrocytes.^135^

The findings from the in vitro studies summarized above can be difficult to interpret in regard to the expected physiological levels of MCFAs and ketone bodies in brain tissue. Although high plasma concentrations of decanoic acid of 50–400 µmol·L^–1^ have been reached in people eating MCTs, it is doubtful that these levels can be reached in the extracellular fluid of the brain and within cells. It should also be considered that MCFAs are mostly bound to albumin in blood^136^ and inside cells are likely bound to other proteins. Thus, the free active concentrations are expected to be low. On the other hand, it is encouraging that several laboratories have described similar protective effects on mitochondria and antioxidant actions of decanoic acid in vivo and in vitro, and there is evidence that antioxidant defense mechanisms can play a role in anticonvulsant activity.^30^, ^35^, ^71^ More studies are needed to fully understand anticonvulsant and protective mechanisms of action of MCFAs.

## 6 OTHER POTENTIAL BENEFICIAL EFFECTS OF MCTs

MCT add‐on diets appear to have benefits beyond epilepsy, by potentially improving functions of the central nervous system and overall metabolism. Here, we provide a few references to recent studies and review articles, as these potential protective effects of MCTs are expected to also benefit people and dogs with epilepsy. Age‐associated cerebral glucose hypometabolism has been reported in rodents, dogs, monkeys, and people, which may contribute to age‐dependent cognitive decline, cognitive impairment, and Alzheimer disease.^137^, ^138^, ^139^, ^140^, ^141^, ^142^ Some evidence suggests that mixed MCTs improve cognition in rodents, dogs, and people with mild cognitive impairments and conditions similar to Alzheimer disease.^68^, ^69^, ^117^, ^143^, ^144^ Neuroprotective effects have been reported in rodent models of Alzheimer disease and Parkinson disease and also with trioctanoin (tricaprylin) in an amyotrophic lateral sclerosis mouse model.^70^, ^145^ There are some indications that add‐on MCTs may be anxiolytic. Feeding a 5% MCT diet to anxious rats reduced anxietylike behaviors and enhanced social competitiveness.^116^ Similarly, there was less stranger‐directed fear in dogs fed a 5.5E% MCT diet.^100^ Finally, several studies have indicated that MCTs may also be beneficial for metabolic syndrome, diabetes, and obesity.^74^, ^146^

## 7 CONCLUSION AND FUTURE DIRECTIONS

Some epilepsies show perturbed utilization of glucose and energy production in epileptogenic brain areas. As most energy in the brain is needed to maintain ion balances and regulate glutamate transport and metabolism, it is likely that energy deficiencies can contribute to hyperexcitability and seizures. Thus, auxiliary brain fuels such as MCFAs and/or ketones are indicated to improve ATP production. Taking the studies discussed above together, there is evidence that dietary inclusion of MCTs provides MCFAs as well as C4 ketone bodies for direct metabolism in the brain without the need of carbohydrate restriction. Also, several studies showed that MCTs improved mitochondrial function and antioxidant defense, and were effective against seizures in rodent models and dogs as well as adults and children with epilepsy. Direct effects of MCFAs potentially together with ketone production are likely to underlie the symptomatic changes in epilepsy, but more studies are needed to confirm these mechanisms. Based on the available studies, it is still unclear which MCT formulations are ideal to prevent seizure generation in people and in dogs. However, decanoic acid and MCTs containing this MCFA seem to play an important role and were more efficacious in in vitro and in vivo rodent models. On the other hand, some people with epilepsy had tolerability issues with add‐on MCTs, so it will be important to develop MCT formulations with higher tolerability.

People with epilepsy and owners of dogs with epilepsy are looking for new epilepsy management options with novel mechanisms of actions without the cognitive side effects often associated with ASDs. Dietary add‐on MCTs are safe and tolerated when emulsified in food and titrated up slowly. To date, neither central nervous system and cardiovascular side effects nor teratogenicity have been observed in rodents, dogs, and humans. Because MCTs have few serious side effects compared to antiseizure medication, use of MCTs may be managed more easily via phone or Skype consultations. This is different from many current ASDs that require closer supervision and sometimes drug monitoring.

In summary, there is now new evidence across species for the efficacy of MCTs for epilepsy management. Direct and indirect as well as metabolic and antioxidant effects of MCFAs possibly together with those of C4 ketone bodies are likely to underlie the anticonvulsant mechanisms of MCTs. For now, more and larger clinical trials are needed especially in humans to find out whether the effects seen in the small trials conducted so far can be replicated in larger cohorts. Randomized controlled studies with sufficient numbers of human patients based on power calculations including the evaluation of confounding factors, such as consumed food, are needed. It will be important to determine the ideal compositions and doses of MCTs as well as the types of epilepsy in children, adults, and dogs that respond best.

## CONFLICT OF INTEREST

F.Y.H. has no conflicts of interest. H.A.V. received funding from the American Kennel Club, American Health Foundation (grant 2252), BBSRC (BB/P001874/1), and Nestlé Purina PetCare to perform clinical trials for MCT‐enriched dog diets for epilepsy and its comorbidities cognitive dysfunction and fear/anxiety behavior. Furthermore, Nestlé Purina PetCare was the industrial partner in a CASE BBSRC PhD studentship (BB/J012491/1) investigating metabolic profiles of dogs receiving MCT diets. H.A.V. has received funding for speaking engagements from Nestlé Purina PetCare. B.Z. and Y.P. report US Patent 9789079. B.Z., L.C.‐S., and Y.P. are employed within the R&D Department of Nestlé Purina PetCare to conduct nutrition research for potential use in future commercial applications. G.R. was employed within the European team of Nestlé Purina PetCare, communicating on scientific studies and products, and also organizing clinical trials of products. K.B. has received consulting fees from Nestlé Purina PetCare for her expertise during the preparation of the manuscript, but remains independent from Nestlé Purina PetCare as last and ultimately responsible author of the review. We confirm that we have read the Journal's position on issues involved in ethical publication and affirm that this report is consistent with those guidelines.
